# Variations in exons 11 and 12 of the multi-pest resistance wheat gene *Lr34* are independently additive for leaf rust resistance

**DOI:** 10.3389/fpls.2022.1061490

**Published:** 2023-02-23

**Authors:** Sylvie Cloutier, Elsa Reimer, Bijendra Khadka, Brent D. McCallum

**Affiliations:** ^1^ Ottawa Research and Development Centre, Agriculture and Agri-Food Canada, Ottawa, ON, Canada; ^2^ Morden Research and Development Centre, Agriculture and Agri-Food Canada, Morden, MB, Canada

**Keywords:** *Lr34*, *Puccinia triticina*, leaf rust, additive effects, wheat, *Triticum aestivum*, 3D structure

## Abstract

**Introduction:**

Characterization of germplasm collections for the wheat leaf rust gene Lr34 previously defined five haplotypes in spring wheat. All resistant lines had a 3-bp TTC deletion (null) in exon 11, resulting in the absence of a phenylalanine residue in the ABC transporter, as well as a single nucleotide C (Tyrosine in Lr34+) to T (Histidine in Lr34-) transition in exon 12. A rare haplotype present in Odesskaja 13 and Koktunkulskaja 332, both of intermediate rust resistance, had the 3-bp deletion typical of Lr34+ in exon 11 but the T nucleotide of Lr34- in exon 12.

**Methods:**

To quantify the role of each mutation in leaf rust resistance, Odesskaja 13 and Koktunkulskaja 332 were crossed to Thatcher and its near-isogenic line Thatcher-Lr34 (RL6058). Single seed descent populations were generated and evaluated for rust resistance in six different rust nurseries.

**Results:**

The Odesskaja 13 progeny with the TTC/T haplotype were susceptible with an average severity rating of 62.3%, the null/T haplotype progeny averaged 39.7% and the null/C haplotype was highly resistant, averaging 13.3% severity. The numbers for the Koktunkulskaja 332 crosses were similar with 63.5%, 43.5% and 23.7% severity ratings, respectively. Differences between all classes in all crosses were statistically significant, indicating that both mutations are independently additive for leaf rust resistance. The three-dimensional structural models of LR34 were used to analyze the locations and putative interference of both amino acids with the transport channel. Koktunkulskaja 332 also segregated for marker csLV46 which is linked to Lr46. Rust severity in lines with Lr34+ and csLV46+ had significantly lower rust severity ratings than those without, indicating the additivity of the two loci.

**Discussion:**

This has implications for the deployment of Lr34 in wheat cultivars and for the basic understanding of this important wheat multi-pest durable resistance gene.

## Introduction

Wheat leaf rust is one of the most common and destructive diseases of wheat (McCallum et al., 2016). It is a production problem in nearly all areas where wheat is grown around the world (Huerta-Espino et al., 2011). Control strategies include early seeding and fungicides, but for economic, environmental and sustainability reasons, efforts to counteract this disease have focused mainly on genetic resistance. Major resistance genes (*R*), also referred to as seedling genes or race-specific genes, tend to conform to the gene-for-gene concept of plant disease resistance where the *R* gene recognizes directly or indirectly the product of its avirulence (*Avr*) counterpart (Flor, 1955). Wheat leaf rust genes *Lr1* (Cloutier et al., 2007), *Lr10* (Feuillet et al., 2003), *Lr13* (Hewitt et al., 2021), *Lr21* (Huang et al., 2003) and *Lr22a* (Thind et al., 2017) are typical race-specific genes with nucleotide binding site (NBS) and leucine-rich repeat (LRR) motifs known to be important for plant-pathogen recognition while the more recently cloned *Lr14a* encodes a membrane-bound ankyrin repeat protein (Kolodziej et al., 2021). A large number of leaf rust resistance genes are such typical race-specific *R* genes but race non-specific genes function differently. The latter constitute a subset of adult plant resistance (APR) genes, and confer partial resistance in a race non-specific manner characterized by a slow rusting phenotype.

APR genes become effective as the plant progresses to the adult stage. Some APR genes are race-specific and function similarly to seedling resistance genes, while others are race non-specific and are quantitative or slow rusting in nature. The slow rusting phenotype includes a longer latency period resulting in slower and less severe growth of the pathogen, hence the term partial resistance. Rust pustules are still present on wheat lines that carry these APR genes, but their size and number are reduced and their development is delayed. In hexaploid wheat, *Lr34*, *Lr42*, *Lr46*, *Lr67* and *Lr68* have been described as non-race specific APR genes (Dyck, 1987; Singh et al., 1998; Hiebert et al., 2010; Herrera-Foessel et al., 2011; Herrera-Foessel et al., 2012; Lin et al., 2022). *Lr34* is one of the best and most durable resistance genes for leaf rust, and it also conditions resistance to stripe rust (*Yr18*), stem rust (*Sr57*), powdery mildew (*Pm38*) and other biotrophic diseases such as spot blotch (Dyck, 1987; McIntosh, 1992; Singh, 1993; Spielmeyer et al., 2005; Lillemo et al., 2008; Lillemo et al., 2013). As such, *Lr34*/*Yr18*/*Pm38*/*Sr57*, called *Lr34* for short, should be classified not only as a race non-specific *APR* gene but more accurately as a multi-pathogen resistance gene (Krattinger et al., 2013; Ellis et al., 2014). Leaf tip necrosis (LTN) is an interesting phenotype of *Lr34* (Singh, 1992) that has been used as a selection tool for *Lr34* and other *APR* genes, although *Lr34*-transgenic durum wheat Stewart expressed the leaf rust *Lr34* but not the leaf tip necrosis phenotype (Rinaldo et al., 2017). *Lr46* (*Lr46*/*Yr29*/*Pm39*/*Sr58*/*Ltn2*) and *Lr67* (*Lr67*/*Yr46*/*Pm46*/*Sr55*/*Ltn3*) have also been described as multi-pathogen resistance genes that similarly produce an LTN phenotype (Lagudah et al., 2007; Herrera-Foessel et al., 2011).

*Lr34* (Krattinger et al., 2009), *Lr42* (Lin et al., 2022) and *Lr67* (Moore et al., 2015) have been cloned. *Lr34* encodes an ATP-binding cassette (ABC) transporter of the ABCG subfamily with two transmembrane (TMD) and two cytosolic nucleotide-binding domains (Krattinger et al., 2009). *Lr67* encodes a hexose transporter with an affinity for glucose; susceptible and resistant isoforms of *Lr67* differ by two amino acid residues (Moore et al., 2015). These genes are not only structurally different from known *R* genes, but they encode proteins of different classes. Interestingly, *Lr42* is a nucleotide-binding site leucine-rich repeat (NLR) gene, but it is not strictly a seedling gene because it confers broad effectiveness against all races at both seedling and adult plant stages (Lin et al., 2022).

Broad spectrum resistance, i.e., little or no race-specificity, is often observed when a gene is brought into a gene pool from a distant relative (Ellis et al., 2014). This is not the case for *Lr34+* (the resistant allele), whose origin post-dates the formation of hexaploid wheat (Krattinger et al., 2011; Krattinger et al., 2013; Dakouri et al., 2014). *Lr34* is located on *Triticum aestivum* chromosome 7D. Independent haplotyping of several hundred accessions of *Aegilops tauschii* (D genome ancestor) revealed that only the *Lr34*- haplotype H2 was present in this gene pool, indicating that the susceptible *Lr34*- allele was the ancestral gene and that the H1 haplotype (*Lr34*+) arose after the interspecific crosses between the *T. turgidum* and *Ae. tauschii* progenitors that produced *T. aestivum* (Krattinger et al., 2011; Krattinger et al., 2013; Dakouri et al., 2014). *Lr34* has orthologs in rice and sorghum, both of which correspond to the susceptible allelic form, and is not present in maize, *Brachypodium*, barley and rye, where it is suggested to have been deleted (Krattinger et al., 2013). In bread wheat, homoeologs are present on chromosomes 7A and 4A but only the 4A homoeolog is putatively functional (Kang et al., 2011).

A total of five haplotypes (H1-H5) have so far been described in spring wheat (Krattinger et al., 2009; Lagudah et al., 2009; Dakouri et al., 2010; Dakouri et al., 2014) and an additional two in winter wheat (Lagudah et al., 2009; Cao et al., 2010). In spring wheat, the resistant (*Lr34*+) haplotype H1 is null/C for the mutation sites in exons 11/12 and the known susceptible (*Lr34*-) haplotypes H2-H4 are all TTC/T. The susceptible haplotypes H2, H3 and H4 differed by the presence of single nucleotide variants in intron 4 and exon 10 (Dakouri et al., 2010; Dakouri et al., 2014). In a germplasm collection of 700 accessions, 698 belonged to the main H1-H4 haplotypes but accessions Odesskaja 13 and Koktunkulskaja 332 had a rare null/T haplotype at exons 11/12, corresponding to haplotype H5 (Dakouri et al., 2010; Dakouri et al., 2014). These two accessions had an intermediate leaf rust phenotype in the field and it was not possible to categorically determine whether either or both mutations in exons 11 or 12 imparted resistance to leaf rust.

It was originally believed that only the mutation in exon 11 was needed to recover full LR34 functionality (Chauhan et al., 2015). However, it is still unclear whether both mutations contribute to the functionality. Here, recombinant inbred line (RIL) populations from Odesskaja 13/Thatcher, Odesskaja 13/Thatcher-*Lr34* near-isogenic line (NIL) RL6058, Koktunkulskaja 332/Thatcher and Koktunkulskaja 332/RL6058 were developed. The severity of leaf rust infection on these lines in the field was measured in six disease nurseries over three years to determine the functionality of the LR34 proteins as encoded by the three segregating haplotypes present in these populations: null/C, null/T and TTC/T. Here we provide phenotyping data to quantify the contribution of both mutations and use protein structure modeling of LR34 to support our results.

## Materials and methods

### Development of plant populations

**Figure 1:**
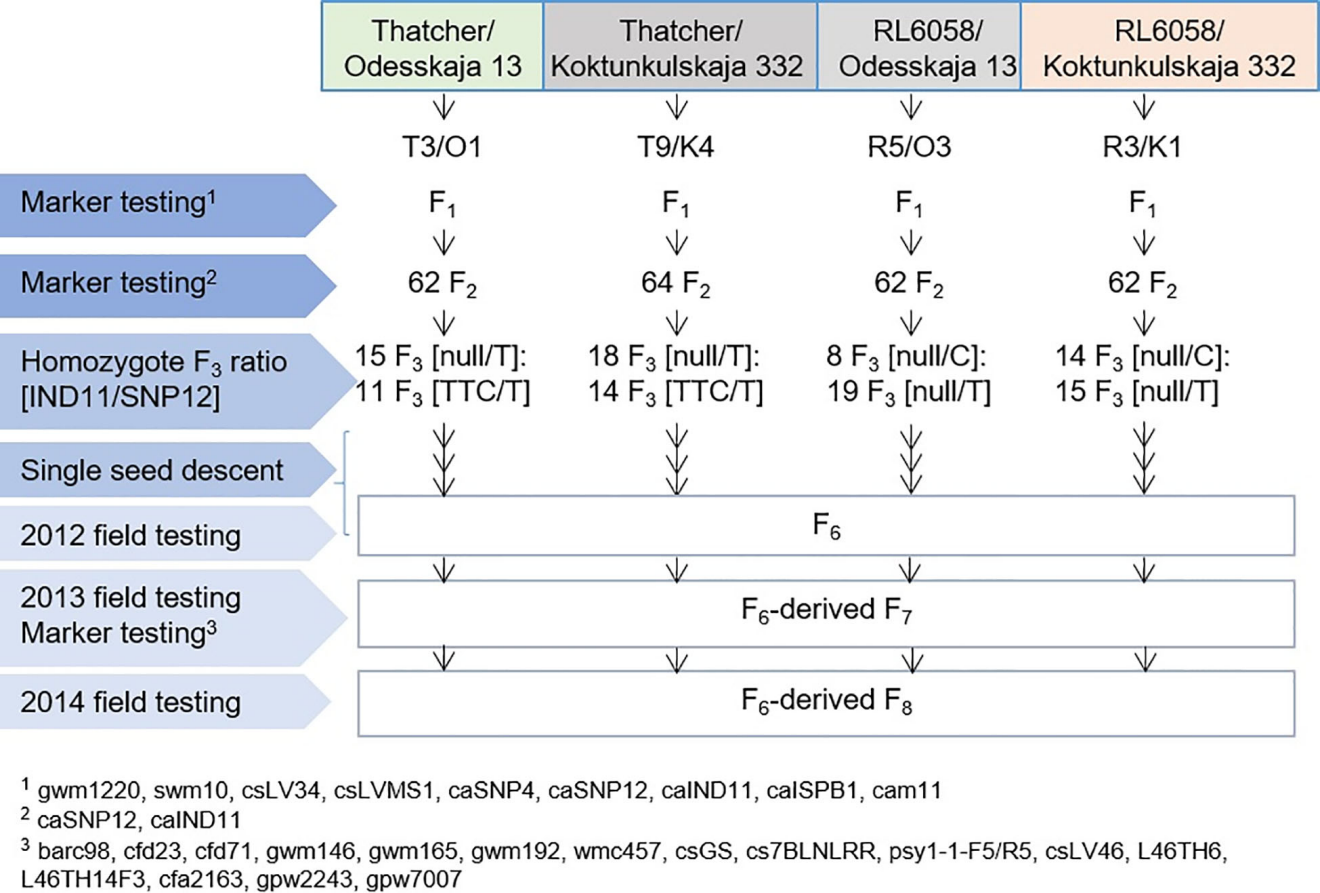
Description of the four populations developed to determine the role of the *Lr34* mutations in exons 11 and 12. Generations tested with molecular markers and phenotypically assessed in the field for severity of leaf rust infection are identified.

Thatcher, a cultivar nearly universally susceptible to wheat leaf rust, has been used to develop a series of NILs, each with a single introgressed leaf rust gene as first described by Dyck and Samborski (1974). RL6058 is the NIL Thatcher*6/PI58548, where PI58548 was the source for *Lr34* (Dyck, 1987). Thatcher (*Lr34*-) has an H2 haplotype (TTC/T for IND11/SNP12), while RL6058 (*Lr34*+) has an H1 haplotype (null/C). We took advantage of the unique *Lr34* allele of Odesskaja 13 and Koktunkulskaja 332 with the rare H5 haplotype (null/T) to develop four RIL populations (**Figure 1**) to demonstrate the leaf rust resistance functionality conferred by each mutation.

Crosses were made between Thatcher and Odesskaja 13, Thatcher and Koktunkulskaja 332, RL6058 and Odesskaja 13 and RL6058 and Koktunkulskaja 332. Genomic DNA was extracted from the F_1_s using the DNEasy kit (Qiagen, Toronto, ON, Canada) as per the manufacturer’s instructions. These F_1_s were screened with nine *Lr34* locus-specific markers to verify heterozygosity across the locus (**Figures 1** and **S1A**). The following nine markers were resolved: gwm1220 (Spielmeyer et al., 2005), swm10 (Bossolini et al., 2006), csLV34 (Lagudah et al., 2006), csLVMS1 (Spielmeyer et al., 2008), and caSNP4, caSNP12, caIND11, caISBP1 and cam11 (Dakouri et al., 2010). Four F_1_ plants, one for each cross, were selected, and selfed F_2_ seeds were harvested. Between 62 and 64 F_2_ plants were grown from each cross. In order to select only homozygous lines, these F_2_s were tested with the caIND11 and caSNP12 markers (**Figure S1B**). Marker caIND11 is co-dominant and the selection of lines homozygous for this mutation was possible using the marker. However, SNP12 (Dakouri et al., 2010) is a dominant marker; hence, it was not possible to distinguish homozygous SNP12-C from heterozygous SNP12-C/T in the RL6058 crosses. To select homozygous SNP12-C and homozygous SNP12-T F_2_s from the two RL6058 crosses, the region spanning exons 10 to 12 was PCR-amplified and sequenced (**Figure S2**). Using this combined maker information, 114 fixed F_2_s (26-32 per cross) were identified and further selfed through to the F_6_ generation by single seed descent (SSD).

### Leaf rust field tests

All 114 RILs from the four crosses were grown in the field in six nurseries over three years. In 2012 (F_6_) and 2013 (F_7_), lines were grown in Winnipeg and Portage La Prairie, Manitoba, Canada, while in 2014 (F_8_), they were grown in Ottawa, Ontario and Morden, Manitoba, Canada. The RIL and parental lines were grown in hills or short 1m-rows spaced every 30 cm, where every five hills or rows were interspersed with the susceptible variety Morocco or a mixture of susceptible lines that served as spreader rows. Due to low seed availability in 2012 and 2013, a completely randomized experimental design with single replicates was used while two replicates were used in 2014. The entire field was inoculated with a mixture of races representative of the *P. triticina* population found in western Canada in the previous year (Dakouri et al., 2013), except for Ottawa in 2014, where the rating was based on natural rust infection. Rust severity ratings and reaction types were recorded using a modified Cobb scale (Peterson et al., 1948) as previously described (Dakouri et al., 2013). An analysis of variance was performed using SAS (SAS Institute Inc., Cary, NC) PROC GLIMMIX, considering replications within site-years as random and genotypes as fixed variables and using a beta distribution in the link. Ratings of the progeny from the Odesskaja 13/Thatcher and Odesskaja 13/RL6058 were combined for the analysis to allow a direct comparison of all possible genotypic classes. The same was done for the Koktunkulskaja 332 progeny. Independent comparisons between contrasting exons 11 and 12 genotypes were performed using least square mean (LSMEANS) differences (*P*<0.05). Comparisons between the haplotype classes null/C, TTC/T and null/T, and these classes with and without the marker for *Lr46* in the Koktunkulskaja 332 progeny, were made using LSMEANS differences (*P*<0.05) with the Tukey-Kramer multiple range adjustment.

### Leaf rust seedling tests

Seedling tests were performed on Odesskaja 13 and Koktunkulskaja 332 using ten different virulence phenotypes of *Puccinia triticina* (BBBD, MBDS, MGBJ, TJBJ, TDBG, MBRJ, PBDQ, THMJ, TNRJ, and TCRJ - nomenclature according to Long and Kolmer (1989)). Odesskaja 13 and Koktunkulskaja 332 were susceptible to all these races, except Koktunkulskaja 332 which was resistant to BBBD (Dakouri et al., 2013). The progeny lines from the four crosses were tested at the seedling stage as previously described (McCallum et al., 2011) to verify the rust resistance phenotype to virulence phenotype BBBD of *P. triticina*, as previously determined (Dakouri et al., 2013). Briefly, infection types produced on the infected lines were rated 12 days after inoculation. Interactions that produced infection types “;” (hypersensitive flecks), “1” (small uredinia with necrosis), and “2” (small- to medium-size uredinia with chlorosis) were considered resistant responses, and those that produced infection types “3” (medium-size uredinia without chlorosis or necrosis) and “4” (large uredinia without chlorosis or necrosis) were considered susceptible.

### Molecular marker testing for other adult plant resistance (APR) genes

To test for the possible presence of *APR* genes other than *Lr34*, the previously reported molecular markers linked to *Lr46*, *Lr67*, *Lr68* and *Trp1* were assessed. Genomic DNA was extracted from single F_6_-derived F_7_ plants of all 114 lines, Odesskaja 13, Koktunkulskaja 332, RL6058 and Thatcher, as well as the following lines known to carry one or more of these *APR* genes: Lalbahadur (Pavon 1B), RL6077, Parula, Toropi 6.3 and Glenlea. In addition, the following markers linked to *Lr67* were tested: barc98, cfd23, cfd71, gwm165, gwm192 and wmc457 (Hiebert et al., 2010; Herrera-Foessel et al., 2011) (**Figure S1C**). Several markers were tested as follows: gwm146, csGS, cs7BLNLRR (Herrera-Foessel et al., 2012) and psy1-1-F5/R5 (Pozniak et al., 2007) for *Lr68*, csLV46 (Evans Lagudah, personal communication) for *Lr46*, and cfa2163, gpw2243 and gpw7007 (Rosa, 2013) for *Trp-1*.

### Structural models of LR34 proteins

RoseTTAFold (Baek et al., 2021) and AlphaFold2 (Jumper et al., 2021) were used to predict the three-dimensional (3D) structure of the LR34 isoforms corresponding to the haplotypes studied herein. The protein sequences of LR34+ (Accession ACN41354) and LR34- (Accession ACL36478) served as inputs for structural prediction. The quality of RoseTTAFold generated models were characterized using a confidence score (1 good, 0 bad), which corresponds to the average pairwise TM-score of the top ten Rosetta scoring models (Baek et al., 2021). AlphaFold2 (AF2) predicted models were also assessed using the pLDDT (predicted Local Distance Difference Test) score (Mariani et al., 2013). AF2 produces pLDDT, a per residue confidence score ranging from 1 to 100, where values >80 indicate high confidence of the residue structure in the protein regions. Therefore, the best-ranked model of LR34 with the highest overall pLDDT and confidence score (0.75) was selected for structural analysis. Molecular graphics and structural model analyses were performed using PyMOL (The PyMOL Molecular Graphics System, Version2 Schrödinger, LLC) and UCSF ChimeraX (Pettersen et al., 2021).

## Results

### Assessment of rust severity in the field

**Figure 2:**
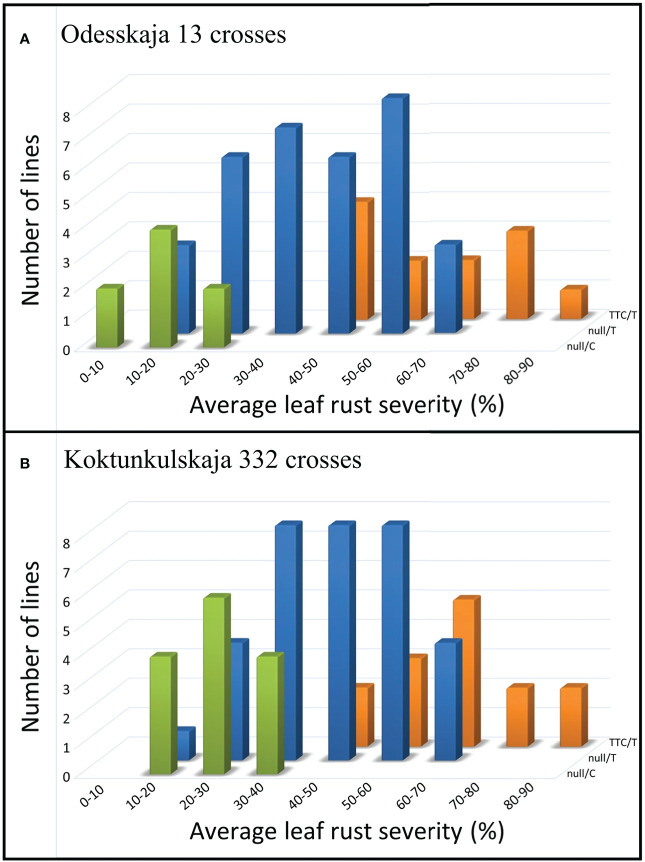
Average leaf rust severity (%) of lines from the Odesskaja 13 crosses **(A)** and the Koktunkulskaja 332 crosses **(B)** categorized according to their *Lr34* haplotypes as defined by their genotype at exons 11 and 12, namely null/C, null/T and TTC/T. Populations were tested in six environments over three years.

**Figure 3:**
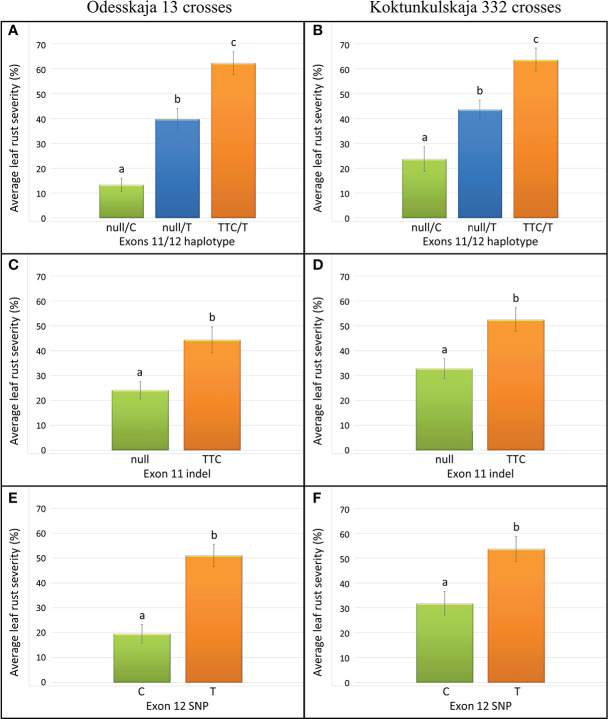
Leaf rust severity (%) of lines from the Odesskaja 13 crosses **(A, C, E)** and the Koktunkulskaja 332 crosses **(B, D, F)** obtained by averaging over all environments and years and classified according to their genotype at exons 11/12 **(A, B)**, exon 11 only **(C, D)** and exon 12 only **(E, F)**. Letters above the error bars indicate statistical significance (*P* < 0.05).

The five previously described *Lr34* haplotypes (Dakouri et al., 2014) are summarized for reference and nomenclature purposes in **Table S1**. There were 26 RILs generated from the cross Thatcher/Odesskaja 13 (14 null/T and 12 TTC/T), 32 from Thatcher/Koktunkulskaja 332 (18 null/T and 14 TTC/T), 27 from RL6058/Odesskaja 13 (8 null/C and 19 null/T) and 29 from RL6058/Koktunkulskaja 332 (14 null/C and 15 null/T) (**Figure 1**). Typical *Lr34*-type rust infection symptoms for the susceptible line Thatcher and its near isogenic line RL6058 (Thatcher-*Lr34*) shown in **Figure S3** represent the symptoms observed in the field nurseries. Rust severity evaluated in the fields was low for the null/C RILs ranging from 5 to 35%; null/T RILs had intermediate rust severity in the range of 12 to 69%; while TTC/T RILs were highly susceptible to leaf rust, displaying rust severity ranging from 45 to 81% (**Figure 2**, **Table 1** and **Supplementary Data Table S2**). Comparisons of the three classes defined by the combination of the two mutations were all statistically significant (*P*<0.05) for both the Odesskaja 13 and the Koktunkulskaja 332 crosses (**Figures 2**, **3**). In the crosses with Odesskaja 13, progeny with TTC/T were susceptible with an average severity of 62.3%, null/T lines averaged 39.7% severity, while null/C lines were highly resistant, averaging 13.3% severity (**Figure 3A** and **Table 1**). Similarly, in the Koktunkulskaja 332 crosses, TTC/T progeny averaged a severity rating of 63.5%, null/T progeny of 43.5% and null/C progeny of 23.7% (**Figure 3B** and **Table 1**). Individually considered, exon 11 “TTC” RILs averaged 52.5% rust severity in the Odesskaja 13 crosses and were significantly (*P*<0.05) more susceptible than exon 11 “null” RILs, which averaged only 32.8% (**Figure 3C**). Similarly, for the Koktunkulskaja 332 crosses, exon 11 “TTC” RILs averaged 44.4% rust severity and were significantly more susceptible than exon 11 “null” RILs at 24.1% (**Figure 3D**). For exon 12, “T” RILs in the Odesskaja 13 crosses averaged 53.4% rust severity compared to 31.8% for RILs with exon 12 “C” (**Figure 3E**). In the Koktunkulskaja 332 crosses, these numbers were 51.1% and 19.6% and were also significant (**Figure 3F**).

**Table 1:** Leaf rust severity ratings (%) of the Odesskaja 13 and Koktunkulskaja 332 derived near-isogenic line populations based on their *Lr34* haplotype.

	Average leaf rust severity (%)		
	H1	H5	H2
	null/C	null/T	TTC/T
Odesskaja 13 crosses^1^	13.3	39.7	62.3
Koktunkulskaja 332 crosses^2^	23.7	43.5	63.5
Range – all crosses	5-35	12-69	45-81

^1^Thatcher/Odesskaja 13 and RL6058/Odesskaja 13.

^2^Thatcher/Koktunkulskaja 332 and RL6058/Koktunkukskaja 332.

### Leaf rust seedling tests

Odesskaja 13 was postulated to have no leaf rust seedling resistance genes while Koktunkulskaja 332 was resistant only to race BBBD (Dakouri et al., 2013). The progeny lines from the Koktunkulskaja 332/Thatcher and Koktunkulskaja 332/RL6058 crosses were tested for seedling resistance to BBBD to see if this seedling gene was effective against leaf rust and could contribute to the resistance phenotype measured in the rust nurseries. The segregation of resistant to susceptible in the progenies fit a single gene hypothesis with 28 resistant (“;” or fleck rating) and 31 susceptible (“4” rating). However, there was no difference between the lines that possessed the seedling resistance gene and those that did not in terms of their leaf rust severity ratings, indicating that the seedling gene present in Koktunkulskaja 332 has been overcome and is ineffective. This was expected because Koktunkulskaja 332 was susceptible to nearly all *Puccinia triticina* virulence phenotypes found in Canada. The progeny lines of the Odesskaja 13/Thatcher and Odesskaja 13/RL6058 crosses were also tested and found to all be susceptible to BBBD, as previously reported (Dakouri et al., 2013).

### Additional adult resistance gene postulation

**Figure 4:**
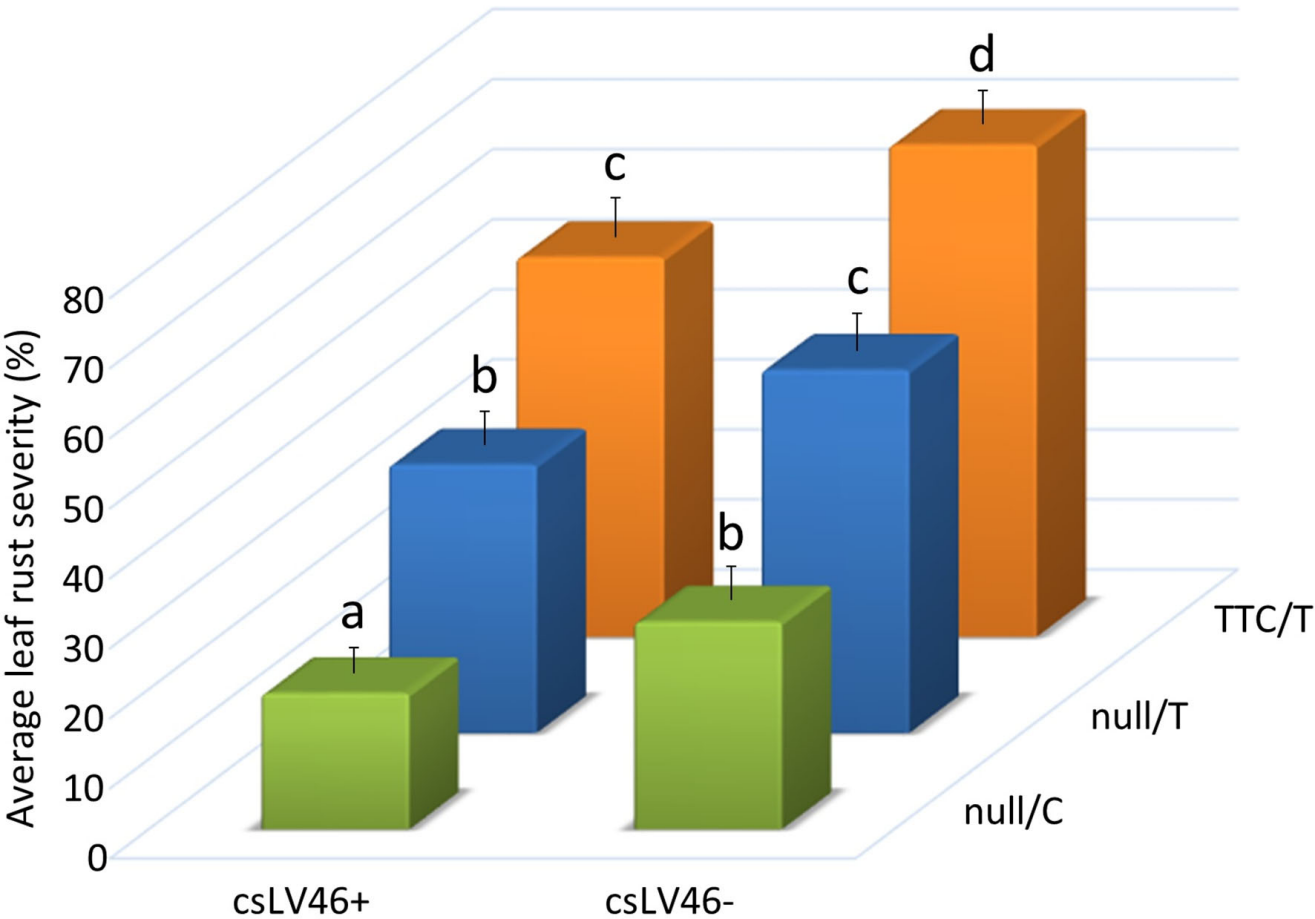
Average rust severity (%) of lines derived from crosses between Koktunkulskaja 332 and either Thatcher or its near-isogenic *Lr34* line RL6058 where the lines are grouped according to their *Lr34* and their csLV46 marker haplotypes. Marker csLV46 is linked to the leaf rust adult plant resistance gene *Lr46*. Letters above the error bars indicate statistical significance (*P* < 0.05).

The potential segregation of other *APR* genes from either Odesskaja 13 or Koktunkulskaja 332 was investigated. *Lr46*, *Lr68* and *Trp1* were previously mapped with various degrees of precision using molecular markers. However, their isolation has not been reported to date (Hiebert et al., 2010; Herrera-Foessel et al., 2011; Rosa, 2013), while *Lr67* has been isolated (Moore et al., 2015). A total of 14 molecular markers for the four additional *APR* genes were resolved on all 114 RILs (**Figure S1C**). Single marker analyses for rust severity indicated that *Lr34* markers were significant in the Odesskaja 13 and Koktunkulskaja 332 crosses, and csLV46 was the only other significant marker and this was observed in five of the eight site-years for the Koktunkulskaja 332 crosses but not for the Odesskaja 13 crosses (**Supplementary Data Tables S3** and **S4**). Marker csLV46 is linked to *Lr46*. Thatcher, RL6058 and Odesskaja 13 all have the ~206 bp *csLV46-* allele, while Koktunkulskaja 332 produced the *csLV46+ Taq*1-restricted cleaved amplified polymorphic sequence (CAPS). To assess the leaf rust resistance associated with the *csLV46* alleles and their interactions with the *Lr34* alleles, the rust severity of the progeny classes obtained from the Koktunkulskaja 332 crosses were compared. Severity averaged 19% for RILs with the H1 *Lr34* haplotype (null/C) and *csLV46+* alleles to 70% for lines with the H2 *Lr34* haplotype (TTC/T) and *csLV46-* (**Figure 4**). In addition, gradients of severity were observed for both genes indicating an additive gene action between *Lr34* and csLV46 where susceptible to resistant haplotypes were in this order: TTC/T > null/T > null/C for *Lr34* and *csLV46-* > *csLV46+* for the csLV46 marker linked to *Lr46* (**Figure 4**).

### Analysis of the predicted structural model of LR34

**Figure 5:**
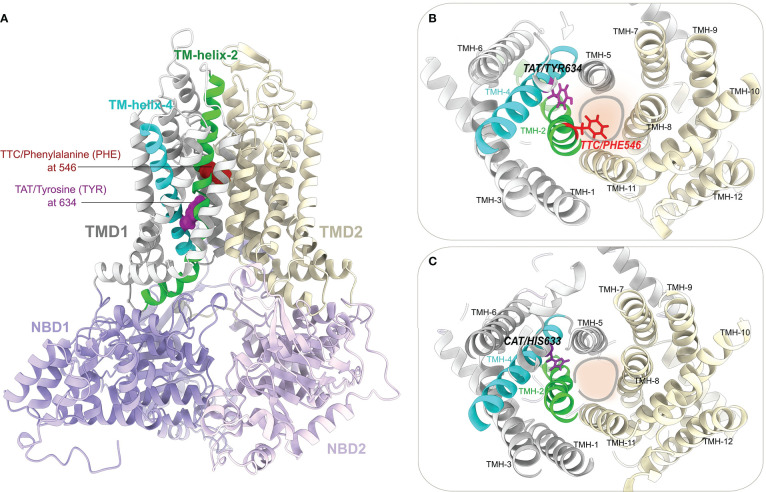
Predicted structural model of the LR34 protein. **(A)** Ribbon representation of the structural model of full-length LR34- generated using RoseTTAFold. The LR34- phenylalanine (PHE 546) located in transmembrane helix-2 (TMH-2), and tyrosine (TYR634) residues located in TMH-4 are displayed as red and magenta colored spheres, respectively. The two transmembrane domains (TMD1 and TMD2) and two nucleotide-binding domains (NBD1 and NBD2) are highlighted and labeled. **(B)** A view from the top (periplasmic) side of the LR34- model shows the arrangement of 12 transmembrane helixes in TMD1 (TMH-1 to 6) and TMD2 (TMH-7 to 12). The PHE546 and TYR634 residues at TMH-2 and TMH-4 are located close to the substrate binding sites/channel (marked by a circle). **(C)** Top (periplasmic) view of LR34+ with its histidine residue substitution at 633 (HIS633) in TMH-4 and deletion of PHE546 in TMH-2.

3D structural models of three full-length LR34 proteins representing the resistant H1 haplotype (*Lr34+*), the susceptible H2 haplotype (*Lr34*-) and the intermediate H5 haplotype were generated. **Figure 5A** shows a predicted model of LR34- where the structural domains are labeled and highlighted using different colors. The suggested substrate binding sites/channel is located between the transmembrane domain 1 (TMD1) and TMD2 of LR34. As shown in **Figures 5A, B**, the LR34- protein contains a phenylalanine residue at position 546 (PHE546) in transmembrane helix-2 (TMH-2) and a tyrosine residue at position 634 (TYR634) in TMH-4 of TMD1. The PHE546 and TYR634 residues point their side chains toward the substrate binding/translocation channel, particularly the aromatic side chain of PHE546, which appears to be pore-occluding (**Figure 5B**). **Figure 5C** shows the structural model of LR34+, with its deleted PHE546 at TMH-2 and substitution of tyrosine with histidine at position 633 (HIS634) of TMH-4. To get additional insights into the possible LR34 substrate binding sites, structural comparisons of our predicted LR34 models with the recently available experimentally-solved structures of substrate or ligand-bound ABCG/Pdr5-like ABC transporters were also performed (**Figure S4**).

## Discussion

*Lr34* is a multi-pathogen resistance gene that confers slow rusting resistance in wheat. Its cloning led to several studies about its nature, origin, evolution, distribution, structural organization and functionality (Krattinger et al., 2009). The five *Lr34* haplotypes (H1-H5) reported in spring wheat are defined based on mutations in intron 4 and exons 10, 11 and 12 (Dakouri et al., 2014). Resistant genotypes (*Lr34*+) all have the H1 haplotype, while H2, H3 and H4 haplotype germplasm is susceptible (*Lr34*-) (Dakouri et al., 2010; Dakouri et al., 2014). The common differences between these two phenotypic classes are the mutations in exons 11 and 12. The resistant H1 haplotype is null/C while the susceptible H2, H3 and H4 haplotypes are all TTC/T for exons 11/12 (**Table S1**). Because of the proximity of these two mutations, the vast majority of the accessions (698/700) previously tested were either null/C or TTC/T; hence it was not possible to partition the causal role of these two mutations using this extensive germplasm. However, the H5 haplotype is null/T, a haplotype that could be useful for addressing this question. This could not be clearly addressed by Dakouri et al. (2014) because only two accessions, both with intermediate severity ratings, possessed this haplotype. To confirm each mutation’s contribution to leaf rust resistance, populations that segregated for these different haplotypes were developed. To do so, the null/T haplotype accessions Odesskaja 13 and Koktunkulskaja 332 were crossed to Thatcher and its *Lr34* NIL RL6058, and RIL populations segregating for haplotypes H1, H2 and H5 were developed. The severity of leaf rust infection in the field at two locations per year over three years was then measured. To date, accessions with the TTC/C haplotype have not been identified, and only three of the possible four classes segregated in these crosses.

Our results indicated that exons 11 and 12 mutations independently and additively contribute to leaf rust resistance. H1 haplotype (null/C) RILs were significantly more resistant than H5 haplotype (null/T) RILs which, in turn, were significantly more resistant than H2 haplotype (TTC/T) RILs (**Figures 2**, **3A, B**). The same effect was observed in crosses with Odesskaja 13 and Koktunkulskaja 332 (**Figures 3A, B**), indicating that this is not specific to one genetic background. Overall, the RILs in the Odesskaja 13 crosses were slightly more resistant than their counterparts in the Koktunkulskaja 332 crosses harboring the same haplotypes (**Figures 2**, **3A, B**); differences in their genetic backgrounds being the most likely cause, although this could not be attributed to any specific leaf rust seedling genes based on our screening with ten races. Additionally, when the effects of each mutation were analyzed separately, RILs with the “null” exon 11 allele were significantly more resistant than RILS with the “TTC” allele in both crosses (**Figures 3C, D**). For exon 12, RILs with the “C” allele were significantly more resistant than RILs with the “T” allele (**Figures 3E, F**). Marker csLV46, linked to *Lr46*, segregated in the Koktunkulskaja 332 crosses. Though it was not as effective as *Lr34*, the resistance conditioned by the Koktunkulskaja 332 putative *Lr46* gene was additive to the resistance conditioned by both *Lr34* exon mutations (**Figure 4**).

The TTC to null exon 11 mutation corresponds to the deletion of a phenylalanine residue at position 546 in the first TMD of LR34, and the T to C exon 12 mutation converts a tyrosine into a histidine at position 633, also in the first TMD (Krattinger et al., 2009). Chauhan et al. (2015) transformed barley with *Lr34* constructs called M1 (null/T; equivalent to H5) and M2 (TTC/C; no known haplotype). The M1 transgenic lines displayed leaf rust resistant and LTN phenotypes. In contrast, the M2 transgenic lines were susceptible to rust and showed no LTN, suggesting that, in barley, deletion of the phenylalanine residue was sufficient to provide *Lr34*-based disease resistance, and that the exon 12 encoded tyrosine residue alone was insufficient for leaf rust resistance functionality. Transformation of wheat with the M1 construct also conferred partial resistance in seedling tests. This agrees with our results that support the functional association with the phenylalanine deletion. However, our results demonstrate that both mutations independently contribute to leaf rust resistance in wheat and are additive in the genetic backgrounds of Odesskaja 13 and Koktunkulskaja 332. Because neither variety possesses effective seedling resistance genes, the field severity ratings measured reflect only APR genes.

Gene stacking or pyramiding has long been an effective strategy against wheat leaf rust. Wheat germplasm with three or more seedling resistance genes had significantly lower severity than accessions with fewer genes (Dakouri et al., 2013). APR genes such as *Lr34* were also reported to enhance the effectiveness of other *R* genes (German and Kolmer, 1992; Vanegas et al., 2008). Combining multiple APR genes is also beneficial as lines with multiple APR genes were reported to have near immunity against rusts (Singh et al., 2000). Silva et al. (2015) reported interactions between *Lr68*, *Lr34* and *Sr2* in Uruguay, where they stated that, in their environments, the positive effect of *Lr68* exceeded that of *Lr34*. They hypothesized that the enhanced expression of *Lr34* at colder temperatures might be the reason for their observations. Lillemo et al. (2008) found a non-additive interaction between *Lr34* and *Lr46*, while Herrera-Foessel et al. (2009) reported only a marginal improvement of the gene combination. However, a recent report indicated the additive effect between the two genes (Bokore et al., 2022); the latter being consistent with our findings in the Koktunkulskaja 332 populations, where *Lr34* and an APR gene postulated to be *Lr46* were both effective and additive. Indeed, assuming that lines with csLV46 carry the APR gene *Lr46*, RILs carrying both APR genes displayed significantly lower rust infection severity than RILs with a single APR gene which, in turn, had lower infection ratings than lines with neither gene. The molecular basis for *Lr46* was hypothesized to differ from that of *Lr34*, i.e., the former is not an ABC transporter, an observation that prompted Lagudah (2011) to recommend using the gene combination as a preventative strategy should any of the two mechanisms be overcome. While there is no evidence of either gene being ineffective, our results suggest a certain degree of additivity between the two genes, which could be explained by different modes of action. However, this is not direct evidence because many more alternative scenarios could explain the additivity.

Using the RoseTTAFold and Alphafold2, the 3D structural fold of LR34 was predicted. As suggested, the predicted 3D models of LR34 were found to contain 12 transmembrane helixes, with six in each TMD (Krattinger et al., 2011). The structural locations of the two non-synonymous mutations in LR34+ and LR34- were further analyzed to show that they are located in the transmembrane helix TMH-2 and TMH-4, respectively, in close proximity to the putative substrate binding sites/channel. The aromatic side chain of the phenylalanine (PHE546) residue in LR34- located in TMH-2 appears to create a structural obstacle in the substrate translocation channel (**Figure 5**). The functional significance of phenylalanine residue within transmembrane helixes of membrane transporters is well established, and its role in substrate binding, channel gating and transport activities have been reported (Kuo et al., 2003; Rojas et al., 2007; Madej and Kaback, 2013; Alegre et al., 2016; Ganz et al., 2019; Selvam et al., 2019). The second mutation, histidine (HIS634) in LR34-, replaced by tyrosine (TYR633) in LR34+, in TMH-4 of TMD1, is also located near the substrate binding sites/channel. The tyrosine residues are known to stabilize the positive charges within the membrane electric field and contribute to substrate transport function (Dougherty, 1996; Chen et al., 2000; Wu et al., 2008). Likewise, the histidine residues also serve critical roles in the TMDs of transmembrane proteins (Wiebe et al., 2001; Stewart et al., 2007; Wang et al., 2013). In particular, histidine residues are known to undergo protonation state changes in a pH-dependent manner, resulting in a conformation change in transmembrane helixes, change in pore sizes, and to influence the substrate transport activity (Todt and McGroarty, 1992; Chen et al., 2000; Harrison, 2008; Martfeld et al., 2016). Thus it is reasonable to speculate that the substitution of the tyrosine by histidine in LR34-, may result in improved stability, flexibility, and additional structure conformation changes altering substrate translocation activities. This may explain the functional differences between LR34 of H1, H2 and H5 haplotypes. Additionally, at the sequence level, both the histidine/tyrosine residues at position 633/634 are conserved among all plant LR34 orthologues (Krattinger et al., 2011), implying the functional significance of both tyrosine and histidine residues in TMH-4 of LR34.

*Lr34* encodes an ABCG transporter that confers race non-specific resistance to wheat leaf rust (Krattinger et al., 2009). Arabidopsis PEN3/PDR8, another plant ABC transporter, confers non-host resistance to the barley powdery mildew fungal pathogen *Blumeria graminis* f. sp *hordei*, but fungal hyphae growth and infection were observed in *pen3* mutants (Stein et al., 2006). However, these ABC transporter’s exact mode of action in the disease resistance mechanism remains largely unexplored. One of the functions of ABC transporters is the translocation of one or more substrates across membranes. For instance, in *Nicotiana plumbaginifolia*, NpPDR1, an ABCG protein responsible for resistance to the fungal pathogen *Botrytis cinera*, was shown to transport sclareol and to participate in basal plant defense (Kang et al., 2011). Similarly, in Arabidopsis, ABCG25, ABCG36 and ABCG40 are known to transport multiple substrates (Campbell et al., 2003; Kim et al., 2007; Strader and Bartel, 2009; Kuromori et al., 2010; Lu et al., 2015). The ABCG transporter encoded by *Lr34* was originally hypothesized to either export metabolites that affect fungal growth or to provide resistance through a senescence-like process (Krattinger et al., 2009). More recently, abscisic acid (ABA) has been shown to be a substrate of the *Lr34* encoded ABCG transporter in wheat (Krattinger et al., 2019) and also when it is expressed in barley (Bräunlich et al., 2021). Assuming that this is its only mode of action, our results suggest that both the phenylalanine deletion and the tyrosine to histidine substitution independently modify the protein sufficiently to alter the translocation of ABA. The conformation changes caused by one or the other substitution is sufficient to disrupt its function to a certain degree, and this function is disrupted to a greater extent when both mutations are present. LR34 has also been reported to be involved in phospholipid transport (Deppe et al., 2018) and phenylpropanoid accumulation (Rajagopalan et al., 2020), but these may be indirect effects of LR34-dependent ABA allocation (Banasiak and Jasinski, 2022).

Furthermore, it should be noted that the ATP transporters utilize the energy of ATP to trigger conformational changes in the TMD that consequently permits the transport of molecules across membranes (Hollenstein et al., 2007). This is referred to as the ATP-switch model (Higgins and Linton, 2004). In many cases, the substrate binding channel or sites resides between the transmembrane domains TMD1 and TMD2 (Locher, 2016; Lefevre and Boutry, 2018) but the interactions between domains, the order of the activation steps and their consequences on ABC transporter functionality remains unclear (Rees et al., 2009). Of note, a gain-of-function mutation caused by a single amino acid change was also reported for the Arabidopsis *PDR9* gene, which encodes the ABC transporter ABCG37 that imparts resistance to auxinic herbicides (Ito and Gray, 2006). Despite these similarities, further structural, biochemical and functional studies are necessary to better understand the specific roles of the variant amino acid residues in ABC transporters such as LR34. Using the germplasm developed herein and knowing that ABA is a substrate of the LR34 ABCG transporter enable us to design future experiments that could shed further light on the mechanisms of resistance and susceptibility and on this phenomenon of intragenic additivity. Heterologous expression of *Lr34* in barley demonstrated the conservation of the mechanism between wheat and barley while establishing that *Lr34* in barley imparted resistance against leaf and stem rusts as well as powdery mildew (Risk et al., 2013). While the exact mechanism remains unknown, Risk et al. (2012) suggested that *Lr34* may induce pathogenesis-related gene transcripts but it does not directly regulate them, a hypothesis that seems to have been confirmed by the transcriptomics study of *Lr34* barley transgenic lines (Chauhan et al., 2015).

Here we refer to the phenylalanine deletion (null) in exon 11 and the tyrosine (T) to histidine (C) substitution in exon 12 as two gain-of-function mutations from a phenotypic perspective because they both independently contribute additively to APR resistance to leaf rust. However, knowledge of the substrate(s) of LR34- is lacking. Therefore, it remains unclear whether the LR34- function(s) is altered by the two substitutions in LR34+. In other words, is this an added function or a case of neofunctionalization? This is an interesting question that remains to be elucidated.

In conclusion, the data clearly demonstrated that the two non-synonymous mutations located in exons 11 and 12 of *Lr34* were independently additive to adult plant resistance against leaf rust in wheat. This was observed in independent sets of crosses with both Odesskaja 13 and Koktunkulskaja 332 and did not appear specific to the genetic background. The germplasm developed in this study consists of H1 (null/C), H2 (TTC/T) and H5 (null/T) haplotypes which show degrees of resistance to leaf rust in the following order: H1>H5>H2. Krattinger et al. (2019) and Bräunlich et al. (2021) demonstrated that ABA was the substrate of LR34. The germplasm developed herein could be used to clarify the role of each of the two read mutations in ABA transport. If the premise is that both mutations alter the transport channel and that these structural changes affect the transport of ABA, then this can be evaluated using the germplasm developed herein. For instance, the total and relative concentration of ABA in leaves and changes in ABA fluxes in the seedlings can be measured in each haplotype. These results should also have important implications for wheat breeding because *Lr34* is one of the most widely deployed and durable resistance genes, and it confers resistance against several wheat pathogens.

## Data availability statement

The original contributions and datasets presented in the study are all included in the article/**Supplementary Material**. Further inquiries can be directed to the corresponding authors.

## Author contributions

SC and BM designed the experiment, performed the phenotyping and the data analyses and co-wrote the manuscript. ER made the crosses, performed the molecular analysis and phenotyping. BK did the protein modeling and wrote the relevant sections. All authors read and approved the final version of the manuscript.
