# Supplementary figures and images for: Variations in exons 11 and 12 of the multi-pest resistance wheat gene Lr34 are independently additive for leaf rust resistance
Source: Front Plant Sci. 2023 Feb 23;13:1061490. doi: 10.3389/fpls.2022.1061490 (PMC9995823; doi:10.3389/fpls.2022.1061490)

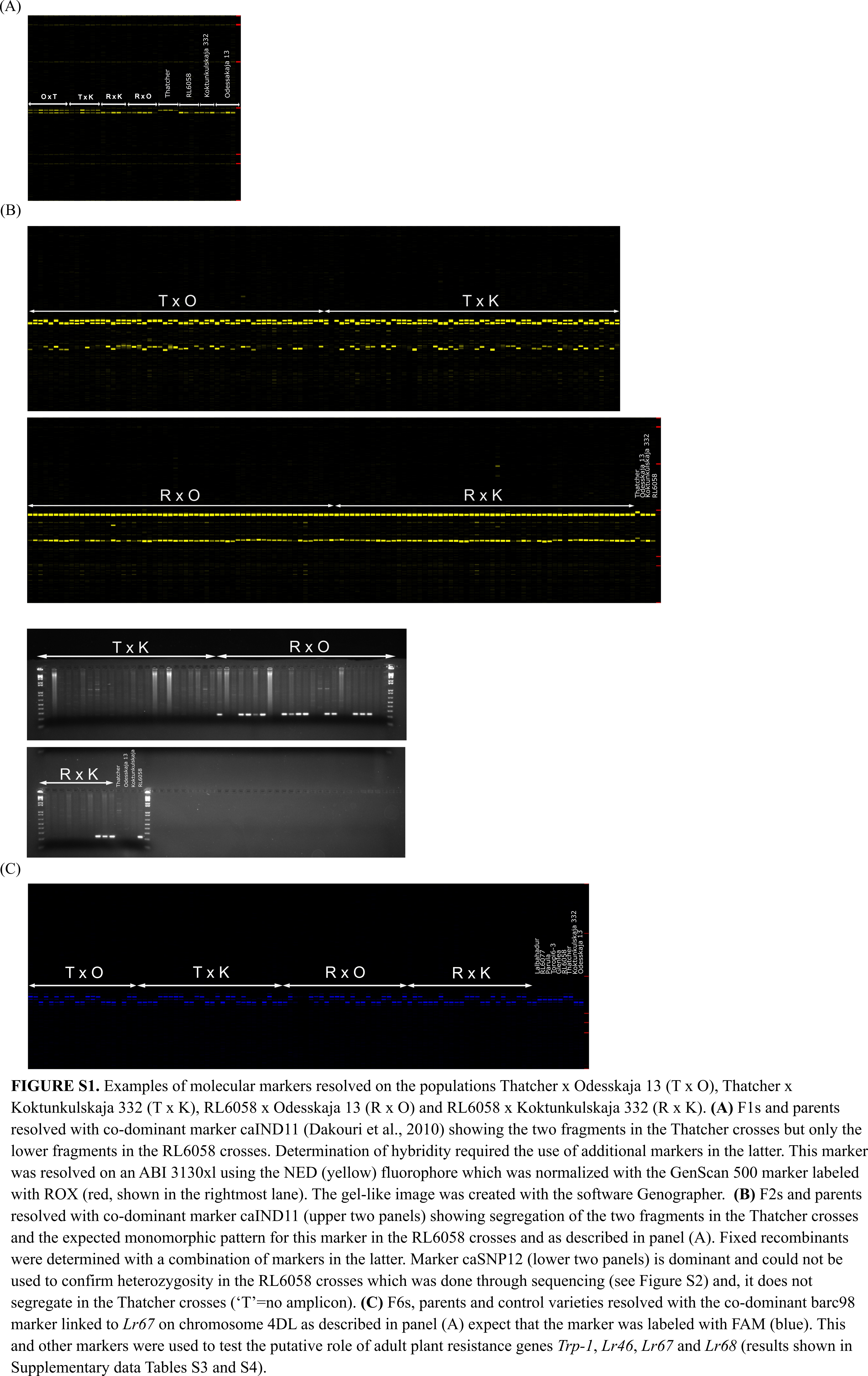

Supplement: Supplementary file 1 [file Image_1.jpeg]

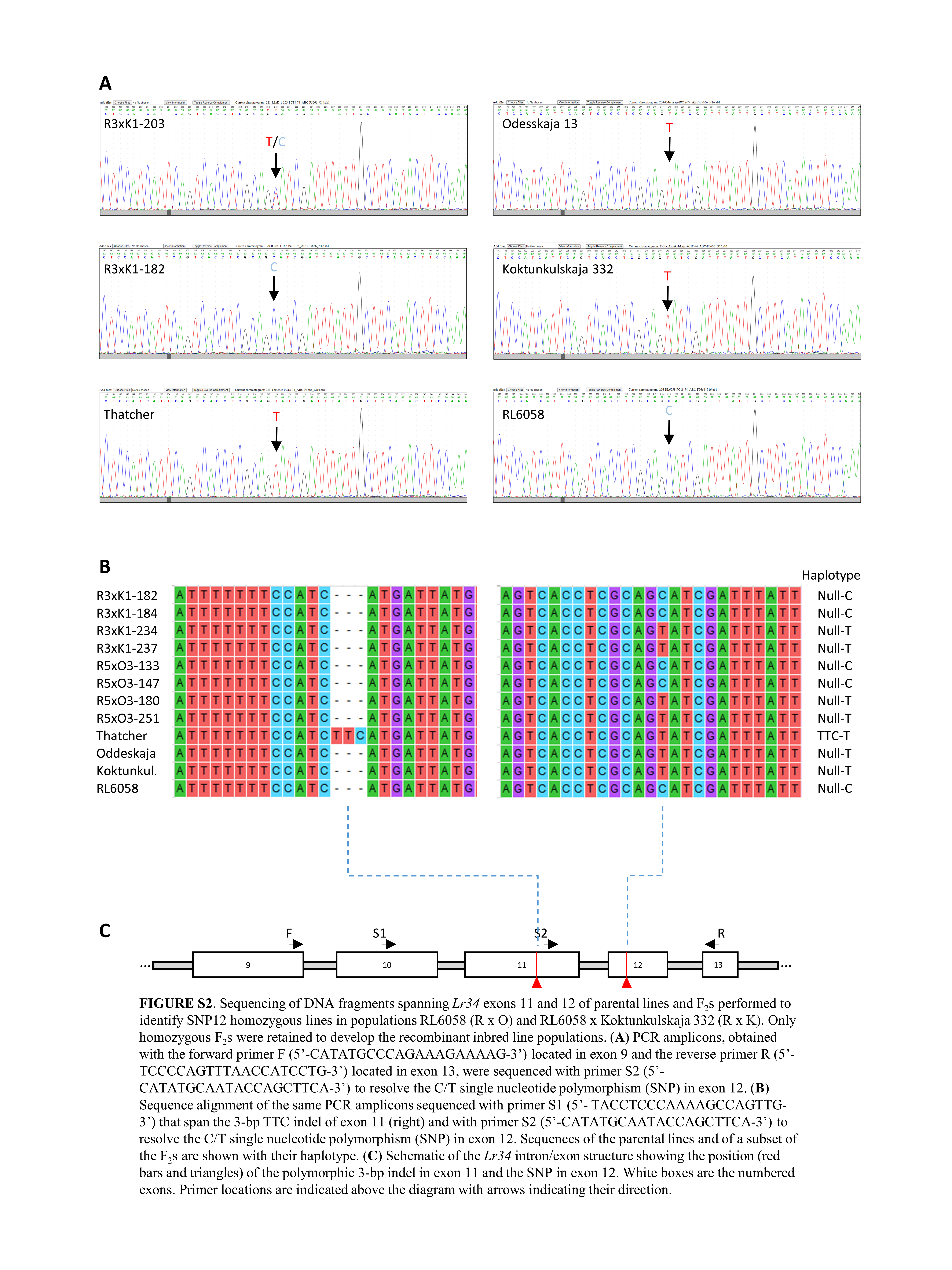

Supplement: Supplementary file 2 [file Image_2.tif]

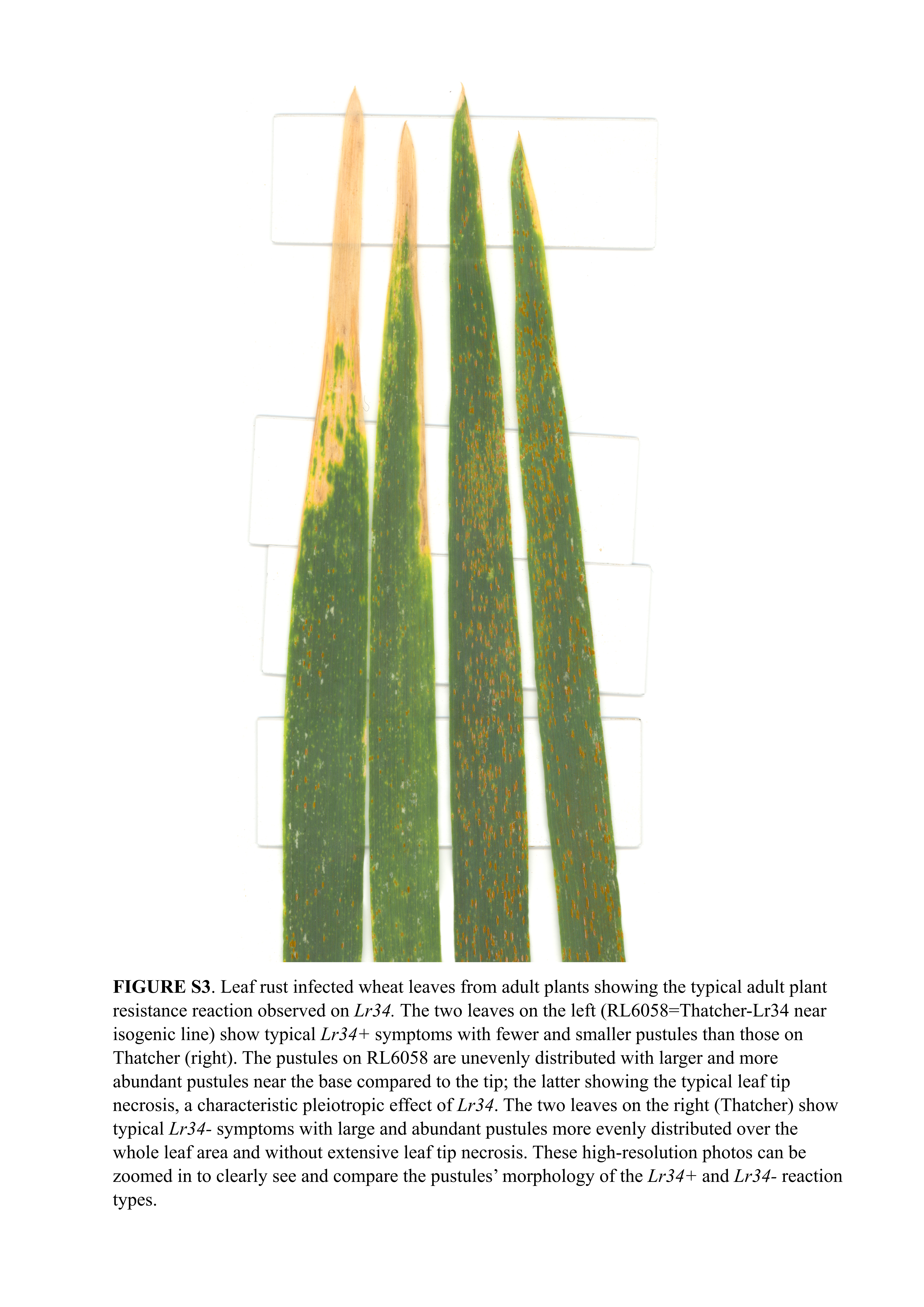

Supplement: Supplementary file 3 [file Image_3.jpeg]

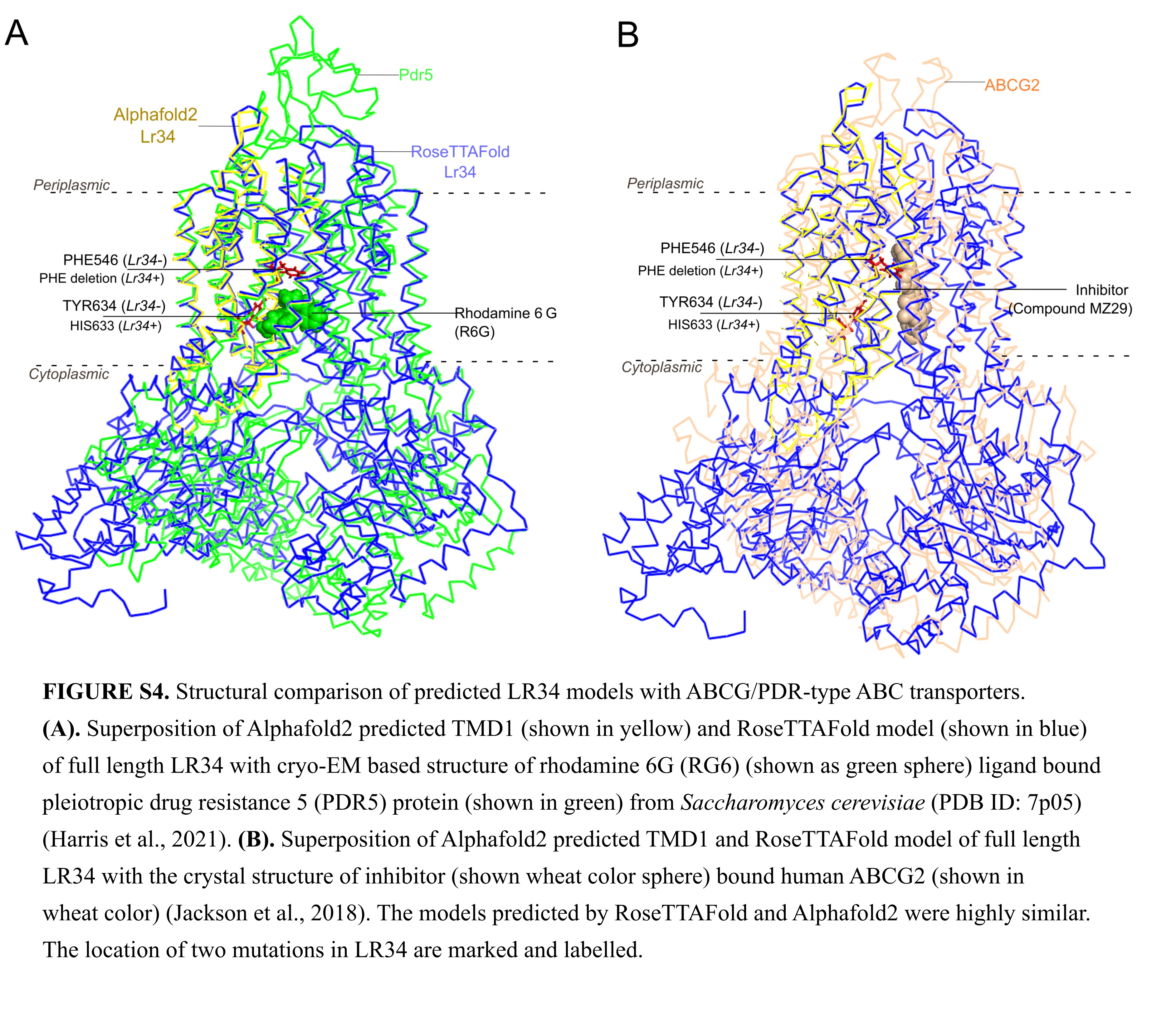

Supplement: Supplementary file 4 [file Image_4.jpeg]
