# Supplementary material for: Variations in exons 11 and 12 of the multi-pest resistance wheat gene Lr34 are independently additive for leaf rust resistance
Source: Front Plant Sci. 2023 Feb 23;13:1061490. doi: 10.3389/fpls.2022.1061490 (PMC9995823; doi:10.3389/fpls.2022.1061490)
Supplement: Supplementary file 5 [file Table_1.docx]

**TABLE S1.** Genotypes at four genic locations within *Lr34* defining five main haplotypes, their frequency in a world germplasm collection of 310 spring wheat accessions and their predicted phenotype based on field evaluations (Dakouri et al., 2014)

| Haplotype | caSNP4^a^ | caIND10 | caIND11 | caSNP12 | Total | Phenotype |
| --- | --- | --- | --- | --- | --- | --- |
| H1 | A | null | null | C | 68 | *Lr34*+ |
| H2 | T | null | TTC | T | 155 | *Lr34*- |
| H3 | A | null | TTC | T | 42 | *Lr34*- |
| H4 | T | A | TTC | T | 43 | *Lr34*- |
| H5 | T | null | null | T | 2 | *Lr34*- (?) |

^a^ Marker names from Dakouri et al. (2010)
