# Supplementary material for: Variations in exons 11 and 12 of the multi-pest resistance wheat gene Lr34 are independently additive for leaf rust resistance
Source: Front Plant Sci. 2023 Feb 23;13:1061490. doi: 10.3389/fpls.2022.1061490 (PMC9995823; doi:10.3389/fpls.2022.1061490)
Supplement: Supplementary file 6 [file Table_2.docx]

**TABLE S2.** Disease severity rating recorded for the recombinant inbred line populations described in Figure 1 from multiple locations, years and replicates.

|  |  | **Leaf rust severity (%)^b^** | | | | | | | |
| --- | --- | --- | --- | --- | --- | --- | --- | --- | --- |
|  | **Location:** | **Winnipeg** | **Portage** | **Winnipeg** | **Portage** | **Ottawa** | **Ottawa** | **Morden** | **Morden** |
|  | **Year:** | **2012** | **2012** | **2013** | **2013** | **2014** | **2014** | **2014** | **2014** |
| **RIL ID^a^** | ***Lr34* haplotype** | **Rep 1** | **Rep 1** | **Rep 1** | **Rep 1** | **Rep 1** | **Rep 2** | **Rep 1** | **Rep 2** |
| T3xO1-1 | null-T | 48 | 70 | 55 | 35 | 35 | 15 | 30 | 35 |
| T3xO1-20 | null-T | 30 | 60 | 35 | 5 | 17.5 | 15 | 10 | 10 |
| T3xO1-26 | null-T | 60 | 80 | 47.5 | 80 | 55 | 35 | 10 | 70 |
| T3xO1-30 | null-T | 58 | 80 | 40 | 70 | 25 | 65 | . | . |
| T3xO1-33 | null-T | 58 | . | 55 | 5 | 7.5 | 3 | . | . |
| T3xO1-37 | null-T | 43 | 80 | 22.5 | 40 | 20 | 7.5 | 10 | 5 |
| T3xO1-40 | null-T | 50 | 80 | 72.5 | 40 | 15 | 30 | 15 | 45 |
| T3xO1-43 | null-T | 43 | 15 | 35 | 65 | 40 | 50 | 15 | 10 |
| T3xO1-47 | null-T | 63 | 80 | 70 | 40 | 5.5 | 35 | 25 | 30 |
| T3xO1-5 | null-T | 58 | 80 | 62.5 | 25 | 30 | 55 | 10 | 10 |
| T3xO1-52 | null-T | 58 | 70 | 80 | 5 | . | . | . | . |
| T3xO1-55 | null-T | 53 | 35 | 60 | 10 | 5 | 10 | 5 |  |
| T3xO1-56 | null-T | 55 | 55 | 75 | 30 | 20 | 40 | 10 | 5 |
| T3xO1-7 | null-T | 70 | 80 | 55 | 65 | 55 | 35 | 50 | 20 |
| T3xO1-11 | TTC-T | 58 | 80 | 62.5 | 60 | 30 | 40 | 30 | 35 |
| T3xO1-19 | TTC-T | 43 | 30 | 70 | 60 | 65 | 55 | 35 | 10 |
| T3xO1-24 | TTC-T | 70 | 80 | 87.5 | 80 | 75 | 55 | 80 | 80 |
| T3xO1-32 | TTC-T | 63 | . | 77.5 | 80 | 70 | 60 | 80 | 55 |
| T3xO1-36 | TTC-T | 55 | 70 | 60 | 80 | 80 | 80 | 25 | 25 |
| T3xO1-39 | TTC-T | 25 | 80 | 62.5 | 65 | 20 | 55 | 40 | 15 |
| T3xO1-4 | TTC-T | 78 | 80 | 82.5 | 80 | 90 | 75 | 80 | 80 |
| T3xO1-45 | TTC-T | 13 | 80 | 27.5 | 70 | 70 | 80 | 70 | 45 |
| T3xO1-46 | TTC-T | 68 | 80 | 67.5 | 80 | 70 | 50 | 50 | 70 |
| T3xO1-59 | TTC-T | 40 | 10 | 60 | 80 | 50 | 35 | 45 | 40 |
| T3xO1-60 | TTC-T | 38 | 80 | 55 | 80 | 85 | 85 | 80 | 70 |
| T3xO1-9 | TTC-T | 50 | 80 | 85 | 80 | 70 | 75 | 70 | 80 |
| T9xK4-101 | null-T | 55 | 80 | 72.5 | 70 | 75 | 70 | 15 | 20 |
| T9xK4-104 | null-T | 25 | 80 | 55 | 65 | 60 | 50 | 50 | 60 |
| T9xK4-105 | null-T | 38 | . | 65 | 20 | 40 | 35 | 15 | 20 |
| T9xK4-109 | null-T | 68 | 70 | 70 | 40 | 25 | 45 | 5 | 20 |
| T9xK4-112 | null-T | 60 | 70 | 75 | 15 | 7.5 | 10 | 20 | 10 |
| T9xK4-114 | null-T | 15 | . | 47.5 | 55 | 25 | 30 | 5 | 30 |
| T9xK4-116 | null-T | 63 | 25 | 80 | 80 | 95 | 80 | 70 | 60 |
| T9xK4-119 | null-T | 73 | 80 | 55 | 30 | 12.5 | . | 20 | 25 |
| T9xK4-246 | null-T | 53 | 65 | 82.5 | 35 | 35 | 60 | 20 | 20 |
| T9xK4-64 | null-T | 45 | 5 | 5 | 15 | 10 | 5 | 0 | . |
| T9xK4-67 | null-T | 48 | 80 | 85 | 80 | 75 | 50 | 50 | 45 |
| T9xK4-75 | null-T | 50 | 45 | 52.5 | 15 | 55 | 25 | 5 | 30 |
| T9xK4-80 | null-T | 45 | 80 | 77.5 | 80 | 35 | 60 | 35 | 30 |
| T9xK4-85 | null-T | 68 | 70 | 62.5 | 40 | 30 | 70 | . | . |
| T9xK4-86 | null-T | 58 | 65 | 57.5 | 30 | 60 | 30 | 10 | 10 |
| T9xK4-88 | null-T | 25 | 70 | 82.5 | 70 | 55 | 45 | 25 | 40 |
| T9xK4-92 | null-T | 53 | 60 | 55 | 35 | 30 | 15 | 20 | 30 |
| T9xK4-96 | null-T | 40 | 55 | 57.5 | 15 | 20 | 15 | 5 | 5 |
| T9xK4-106 | TTC-T | 58 | . | 85 | 80 | 90 | 90 | 80 | 80 |
| T9xK4-107 | TTC-T | 78 | 80 | 85 | 80 | 85 | 85 | . | 70 |
| T9xK4-113 | TTC-T | 65 | 80 | 60 | 70 | 80 | 40 | 55 | 35 |
| T9xK4-117 | TTC-T | 70 | 80 | 95 | 30 | 80 | 65 | 10 | 10 |
| T9xK4-62 | TTC-T | 63 | 80 | 65 | 80 | 65 | 90 | 55 | 70 |
| T9xK4-63 | TTC-T | 80 | 80 | 55 | 20 | 50 | 45 | 25 | 25 |
| T9xK4-66 | TTC-T | 33 | . | 80 | 80 | 90 | 65 | 70 | 65 |
| T9xK4-72 | TTC-T | 33 | 40 | 75 | 70 | 60 | 60 | 60 | 45 |
| T9xK4-74 | TTC-T | 88 | 80 | 72.5 | 80 | 50 | 85 | 65 | 55 |
| T9xK4-76 | TTC-T | 38 | 80 | 70 | 80 | 20 | 80 | 80 | 65 |
| T9xK4-84 | TTC-T | 33 | 80 | 67.5 | 75 | 60 | 25 | 10 | 15 |
| T9xK4-87 | TTC-T | 68 | 80 | 52.5 | 70 | 75 | 50 | 50 | 50 |
| T9xK4-93 | TTC-T | 45 | 80 | 67.5 | 70 | 45 | 80 | 50 | 65 |
| T9xK4-99 | TTC-T | 43 | 70 | . | 45 | 70 | . | . | . |
| R5xO3-133 | null-C | 48 | 15 | 52.5 | 20 | 3 | 7.5 | . | 15 |
| R5xO3-147 | null-C | 28 | 15 | 35 | 5 | 1 | 3 | 0 | 10 |
| R5xO3-149 | null-C | 5 | 25 | 27.5 | 5 | 3 | 7.5 | 0 | . |
| R5xO3-155 | null-C | 13 | 5 | 5 | 0 | 12.5 | 1 | 0 | . |
| R5xO3-166 | null-C | 8 | 15 | 5 | 0 | 7.5 | 5.5 | 0 | 0 |
| R5xO3-174 | null-C | 50 | . | 55 | 10 | 7.5 | 12.5 | . | 10 |
| R5xO3-179 | null-C | 38 | 30 | 45 | 5 | 3 | 5 | 5 | 15 |
| R5xO3-252 | null-C | 43 | 20 | 42.5 | 5 | 7.5 | 15 | 5 | 10 |
| R5xO3-122 | null-T | 63 | 80 | 72.5 | 65 | 45 | 30 | 40 | 40 |
| R5xO3-123 | null-T | 80 | 80 | 85 | 60 | 40 | 45 | 60 | 55 |
| R5xO3-128 | null-T | . | . | 72.5 | 80 | 75 | 60 | 60 | 65 |
| R5xO3-129 | null-T | 23 | 80 | 45 | 70 | 35 | 20 | 5 | 15 |
| R5xO3-134 | null-T | 18 | 40 | 27.5 | 5 | 5 | 1 | 0 | 0 |
| R5xO3-135 | null-T | 28 | 70 | 42.5 | 40 | 60 | 10 | 30 | 30 |
| R5xO3-138 | null-T | 53 | 20 | 62.5 | 40 | 3 | 20 | 5 | 20 |
| R5xO3-139 | null-T | 25 | 45 | 37.5 | 35 | 12.5 | 5 | 30 | 20 |
| R5xO3-143 | null-T | 63 | 80 | 50 | 70 | 50 | 65 | 20 | 30 |
| R5xO3-144 | null-T | 63 | 70 | 70 | 10 | 3 | 5 | 15 | 10 |
| R5xO3-154 | null-T | 38 | 80 | 70 | 80 | 75 | 40 | 70 | 45 |
| R5xO3-162 | null-T | 28 | 80 | 67.5 | 30 | 15 | 7.5 | 25 | 30 |
| R5xO3-163 | null-T | 68 | 80 | 60 | 70 | 30 | 25 | 35 | 60 |
| R5xO3-165 | null-T | 18 | 40 | 15 | 30 | 3 | 1 | . | . |
| R5xO3-168 | null-T | 70 | 80 | 62.5 | 10 | 85 | 40 | 5 | 30 |
| R5xO3-169 | null-T | 28 | 15 | 20 | 20 | 3 | 40 | . | 0 |
| R5xO3-171 | null-T | 25 | 10 | 42.5 | 75 | 80 | 65 | 50 | 30 |
| R5xO3-180 | null-T | 43 | 80 | 65 | 55 | 50 | 45 | 25 | 40 |
| R5xO3-251 | null-T | 40 | 70 | 35 | 55 | 35 | 12.5 | 10 | 20 |
| R3xK1-182 | null-C | 23 | 30 | 40 | 5 | 12.5 | 5.5 | 0 | . |
| R3xK1-184 | null-C | 53 | 30 | 72.5 | 30 | . | 15 | 15 | 30 |
| R3xK1-187 | null-C | 48 | 30 | 27.5 | 5 | 10 | 5 | 5 | 5 |
| R3xK1-190 | null-C | 23 | 40 | 30 | 20 | 7.5 | 7.5 | 5 | 5 |
| R3xK1-197 | null-C | 35 | 30 | 50 | 30 | 15 | 10 | 10 | 25 |
| R3xK1-208 | null-C | 38 | 55 | 57.5 | 25 | 7.5 | 7.5 | 5 | 5 |
| R3xK1-213 | null-C | 18 | 55 | 65 | 45 | 35 | 40 | 15 | 10 |
| R3xK1-221 | null-C | 33 | . | 35 | 15 | 30 | 50 | 5 | 5 |
| R3xK1-222 | null-C | 20 | . | 32.5 | 20 | 10 | 15 | 5 | 30 |
| R3xK1-227 | null-C | 55 | 25 | 60 | 40 | 35 | 20 | 10 | 15 |
| R3xK1-230 | null-C | 58 | 30 | 42.5 | 10 | 5 | 10 | 10 | 5 |
| R3xK1-238 | null-C | 23 | 55 | 27.5 | 0 | 20 | 20 | . | 10 |
| R3xK1-256 | null-C | 30 | . | 55 | 20 | 35 | 40 | 20 | 20 |
| R3xK1-257 | null-C | 23 | 40 | 50 | 15 | 15 | 20 | 5 | 15 |
| R3xK1-185 | null-T | 63 | 45 | 80 | 70 | 65 | 50 | 40 | 15 |
| R3xK1-186 | null-T | 32 | 80 | 60 | 70 | 15 | 20 | 30 | 20 |
| R3xK1-189 | null-T | 45 | 80 | 57.5 | 65 | 35 | 45 | 35 | 20 |
| R3xK1-191 | null-T | 38 | 25 | 67.5 | 25 | 30 | 35 | 5 | . |
| R3xK1-193 | null-T | 48 | 25 | 32.5 | 15 | 25 | 40 | 5 | 5 |
| R3xK1-195 | null-T | 65 | 80 | 25 | 80 | 80 | . | . | . |
| R3xK1-201 | null-T | 28 | 80 | 70 | 30 | 50 | 55 | . | 20 |
| R3xK1-205 | null-T | 40 | 40 | 75 | 50 | 70 | 70 | 45 | 40 |
| R3xK1-212 | null-T | 58 | 80 | 77.5 | 55 | 30 | 30 | 50 | 60 |
| R3xK1-219 | null-T | 25 | 80 | 60 | 80 | 40 | 25 | 10 | 50 |
| R3xK1-226 | null-T | 40 | 65 | 50 | 20 | 10 | 5 | 20 | 15 |
| R3xK1-231 | null-T | 40 | 70 | 60 | 40 | 30 | 35 | 15 | 20 |
| R3xK1-233 | null-T | 55 | 80 | 40 | 35 | 3 | 5 | 20 | 25 |
| R3xK1-234 | null-T | 43 | 80 | 55 | 70 | 80 | 25 | 80 | 55 |
| R3xK1-237 | null-T | 50 | . | 72.5 | 45 | 65 | 70 | 0 | 5 |

^a^ The populations were as follows; T3xO1 = Thatcher-3/Odesskaja 13-1, T9xK4 = Thatcher-9/Koktunkulskaja 332-4, R5xO3 = RL6058-5/Odesskaja 13-3, and R3xK1 = RL6058-3/ Koktunkulskaja 332-1.

^b^ ‘.’ = missing data
