# Supplementary material for: Variations in exons 11 and 12 of the multi-pest resistance wheat gene Lr34 are independently additive for leaf rust resistance
Source: Front Plant Sci. 2023 Feb 23;13:1061490. doi: 10.3389/fpls.2022.1061490 (PMC9995823; doi:10.3389/fpls.2022.1061490)
Supplement: Supplementary file 7 [file Table_3.docx]

**TABLE S3.** *P*-values obtained from the single marker analyses of markers linked to adult plant leaf rust (*Lr*) resistance genes *Lr34*, *Lr46*, *Lr67*, *Lr68* and *LrTrp* of leaf rust severity evaluated at eight site-years of progeny from crosses between Thatcher or RL6058 and Odesskaja 13.

| **Marker** | **Gene** | **WPG12^1^** | **POR12** | **WPG13** | **POR13** | **MOR14-1** | **MOR14-2** | **OTT14-1** | **OTT14-2** |
| --- | --- | --- | --- | --- | --- | --- | --- | --- | --- |
| csLV46 | *Lr46* | 0.608^2^ | 0.662 | 0.749 | 0.224 | 0.853 | 0.787 | 0.119 | 0.060 |
| cfd23 | *Lr67* | 0.244 | 0.945 | 0.317 | 0.561 | 0.668 | 0.619 | 0.536 | 0.302 |
| barc98 | *Lr67* | 0.243 | 0.947 | 0.386 | 0.683 | 0.802 | 0.778 | 0.673 | 0.382 |
| cfd71 | *Lr67* | 0.456 | 0.853 | 0.501 | 0.252 | 0.453 | 0.708 | 0.799 | 0.290 |
| wmc457 | *Lr67* | 0.310 | 0.948 | 0.467 | 0.473 | 0.660 | 0.743 | 0.440 | 0.279 |
| gwm165 | *Lr67* | 0.424 | 0.976 | 0.259 | 0.394 | 0.781 | 0.680 | 0.895 | 0.345 |
| gwm192 | *Lr67* | 0.374 | 0.607 | 0.104 | 0.368 | 0.826 | 0.836 | 0.910 | 0.587 |
| gpw7007 | *Trp1* | 0.687 | 0.763 | 0.124 | 0.055 | 0.144 | 0.894 | 0.369 | 0.185 |
| gpw2243 | *Trp1* | 0.892 | 0.500 | 0.105 | 0.139 | 0.310 | 0.880 | 0.655 | 0.875 |
| cfa2163 | *Trp1* | 0.305 | 0.365 | 0.798 | 0.314 | 0.952 | 0.561 | 0.197 | 0.214 |
| csGS | *Lr68* | 0.281 | 0.076 | 0.593 | 0.297 | 0.433 | 0.953 | 0.119 | 0.206 |
| cs7BLNLRR | *Lr68* | 0.281 | 0.076 | 0.593 | 0.297 | 0.433 | 0.953 | 0.119 | 0.206 |
| psy1-1-F5/R5 | *Lr68* | 0.514 | 0.468 | 0.531 | 0.174 | 0.333 | 0.265 | 0.063 | 0.047* |
| gwm146 | *Lr68* | 0.811 | 0.364 | 0.833 | 0.634 | 0.861 | 0.761 | 0.825 | 0.467 |
| caIND11 | *Lr34* | 0.346 | 0.246 | 0.037* | 5.9E-05**** | 2.2E-05**** | 0.007** | 8.0E-05**** | 8.2E-06**** |
| caSNP12 | *Lr34* | 0.007** | 3.7E-06**** | 0.002** | 1.4E-05**** | 0.003** | 0.015* | 7.7E-04*** | 8.0E-04*** |

^1^ Location-year-replication. Locations were WPG: Winnipeg; POR: Portage La Prairie; MOR: Morden; OTT: Ottawa. Years were 2012, 2013, 2014. Replications 1 and 2 only in 2014.

^2^ * P<0.05; ** P<0.01; *** P<0.001; **** P< 0.0001
