# Supplementary material for: Variations in exons 11 and 12 of the multi-pest resistance wheat gene Lr34 are independently additive for leaf rust resistance
Source: Front Plant Sci. 2023 Feb 23;13:1061490. doi: 10.3389/fpls.2022.1061490 (PMC9995823; doi:10.3389/fpls.2022.1061490)
Supplement: Supplementary file 8 [file Table_4.docx]

**TABLE S4.** *P*-values obtained from the single marker analyses of markers linked to adult plant leaf rust (*Lr*) resistance genes *Lr34*, *Lr46*, *Lr67*, *Lr68* and *LrTrp* of leaf rust severity evaluated at eight site-years of progeny from crosses between Thatcher or RL6058 and Koktunkulskaja 332.

| **Marker** | **Gene** | **WPG12^1^** | **POR12** | **WPG13** | **POR13** | **MOR14-1** | **MOR14-2** | **OTT14-1** | **OTT14-2** |
| --- | --- | --- | --- | --- | --- | --- | --- | --- | --- |
| csLV46 | *Lr46* | 0.912^2^ | 0.259 | 0.140 | 0.001** | 0.001** | 0.002** | 0.006** | 0.003** |
| cfd23 | *Lr67* | 0.896 | 0.737 | 0.610 | 0.684 | 0.897 | 0.760 | 0.861 | 0.688 |
| barc98 | *Lr67* | 0.896 | 0.737 | 0.610 | 0.684 | 0.897 | 0.760 | 0.861 | 0.688 |
| cfd71 | *Lr67* | 0.900 | 0.811 | 0.660 | 0.444 | 0.948 | 0.550 | 0.733 | 0.848 |
| wmc457 | *Lr67* | 0.896 | 0.737 | 0.610 | 0.685 | 0.897 | 0.760 | 0.861 | 0.688 |
| gwm165 | *Lr67* | 0.538 | 0.137 | 0.005** | 0.031* | 0.207 | 0.059 | 0.544 | 0.067 |
| gwm192 | *Lr67* | 0.917 | 0.774 | 0.917 | 0.540 | 0.905 | 0.677 | 0.862 | 0.895 |
| gpw7007 | *Trp1* | 0.237 | 0.869 | 0.724 | 0.758 | 0.679 | 0.918 | 0.956 | 0.733 |
| gpw2243 | *Trp1* | 0.597 | 0.841 | 0.679 | 0.857 | 0.323 | 0.400 | 0.543 | 0.934 |
| cfa2163 | *Trp1* | 0.072 | 0.278 | 0.715 | 0.226 | 0.498 | 0.649 | 0.316 | 0.833 |
| csGS | *Lr68* | 0.625 | 0.384 | 0.995 | 0.996 | 0.201 | 0.599 | 0.337 | 0.570 |
| cs7BLNLRR | *Lr68* | 0.625 | 0.384 | 0.995 | 0.996 | 0.201 | 0.599 | 0.337 | 0.570 |
| psy1-1-F5/R5 | *Lr68* | 0.761 | 0.544 | 0.751 | 0.827 | 0.209 | 0.064 | 0.115 | 0.216 |
| gwm146 | *Lr68* | 0.261 | 0.219 | 0.111 | 0.855 | 0.631 | 0.068 | 0.535 | 0.927 |
| caIND11 | *Lr34* | 0.008** | 0.006** | 0.007** | 2.3E-04*** | 1.1E-05**** | 1.7E-05**** | 5.7E-05**** | 6.2E-06**** |
| caSNP12 | *Lr34* | 0.002** | 1.8E-05**** | 6.2E-04*** | 9.3E-06**** | 0.001** | 0.001** | 1.0E-04*** | 1.6E-04*** |

^1^ Location-year-replication. Locations were WPG: Winnipeg; POR: Portage La Prairie; MOR: Morden; OTT: Ottawa. Years were 2012, 2013, 2014. Replications 1 and 2 only in 2014.

^2^ * P<0.05; ** P<0.01; *** P<0.001; **** P< 0.0001
